# Predictive Factors and Value of ypN+ after Neoadjuvant Chemotherapy in Clinically Lymph Node-Negative Breast Cancer

**DOI:** 10.1371/journal.pone.0162616

**Published:** 2016-09-15

**Authors:** Ippei Fukada, Kazuhiro Araki, Kokoro Kobayashi, Tomoko Shibayama, Shunji Takahashi, Rie Horii, Futoshi Akiyama, Takuji Iwase, Shinji Ohno, Kiyohiko Hatake, Yasuo Hozumi, Naohiro Sata, Yoshinori Ito

**Affiliations:** 1 Breast Medical Oncology, Breast Oncology Center, the Cancer Institute Hospital of the Japanese Foundation for Cancer Research, Tokyo, Japan; 2 Medical Oncology, the Cancer Institute Hospital of the Japanese Foundation for Cancer Research, Tokyo, Japan; 3 Division of Pathology, the Cancer Institute Hospital of the Japanese Foundation for Cancer Research, Tokyo, Japan; 4 Division of Pathology, the Cancer Institute of the Japanese Foundation for Cancer Research, Tokyo, Japan; 5 Breast Surgical Oncology, Breast Oncology Center, the Cancer Institute Hospital of the Japanese Foundation for Cancer Research, Tokyo, Japan; 6 Breast Oncology Center, the Cancer Institute Hospital of the Japanese Foundation for Cancer Research, Tokyo, Japan; 7 Department of Hematology and Oncology, the Cancer Institute Hospital of the Japanese Foundation for Cancer Research, Tokyo, Japan; 8 Department of Surgery, Ibaraki Prefectural Central Hospital, Ibaraki, Japan; 9 Department of Gastrointestinal Surgery, Jichi Medical University, Tochigi, Japan; Institut national de la recherche scientifique, CANADA

## Abstract

**Background:**

Pathological complete response (pCR) with neoadjuvant chemotherapy (NAC) has been regarded as a surrogate endpoint for disease-free survival (DFS) and overall survival (OS) of patients with breast cancer. No consensus regarding the definition of pCR has been established; there are several definitions according to a variety of classifications. Eradication of cancer cells in both breast and lymph nodes has been better associated with improved prognosis than in the breast alone. Even in patients diagnosed as having clinically node-negative cancer before NAC, postoperative pathological examination often shows axillary lymph node metastases.

**Patients and Methods:**

Of the 771 patients with breast cancer who underwent NAC in the Cancer Institute Hospital between January 2000 and May 2009, 146 patients preoperatively diagnosed as having node-negative breast cancer were retrospectively evaluated. We have made the definition of clinically lymph node-negative (N0) as follows: first, ultrasonography before NAC did not show any lymphadenopathy. Second, a cytological procedure confirmed negative study for each patient when ultrasonography suggested lymphadenopathy.

**Results:**

The median observation period was 79.7 months, and the median age of the subjects was 51 years. Pathological examination at the time of the surgery showed lymph node metastases (ypN+) in 46 patients (31.5%). Histological therapeutic effects revealed ypT0/is in 9 patients (6.2%) and ypTinv in 137 (93.8%). Multivariate analysis demonstrated that younger age (49>), large tumor size, NG3, and ypN+ were significant poor prognostic factors for DFS (p = 0.020, p = 0.008, P = 0.022 and p = 0.010, respectively). Moreover, ypN+ was the only significant poor prognostic factor for OS (p = 0.022). The predictive factors of ypN+ in clinically lymph node–negative breast cancer were ypTinv (p = 0.036) and the luminal type (HR+ and HER2-) (p = 0.029).

**Conclusion:**

The prognosis of clinically lymph node negative breast cancer depended on ypN+, which was associated with ypTinv and luminal subtype.

## Introduction

Neoadjuvant chemotherapy (NAC) for breast cancer has been performed mainly in locally advanced cancer or inflammatory cancer, aimed at down-staging. Over the last two decades, pre- and post-operative chemotherapy for early breast cancer has shown similarly favorable overall survival (OS) and disease-free survival (DFS) rates [1, 2]. In patients in whom pCR was achieved after NAC, both the OS and DFS rates were favorable, and NAC increased the rate of breast-conserving surgery [1]. At present, NAC is widely performed in general clinical practice to improve the breast preservation rate and is used as a drug sensitivity test to evaluate the effects of chemotherapy.

In many recent studies, pCR rates associated with NAC have been regarded as a surrogate endpoint for DFS and OS [3, 4]. The US Food and Drug Administration has recently attached great importance to the pCR rate in deciding whether to approve new anti-cancer agents. However, the definition of pCR has tended to vary in each study. Von Minckwitz et al. investigated the association between the prognosis and various definitions of pCR. They reported that pCR defined as no invasive and no *in situ* residuals in breast or nodes could best discriminate between patient outcomes [5]. In a responder analysis, Collaborative Trials in Neoadjuvant Breast Cancer (CTNeoBC) compared the three most commonly used definitions of pCR (ypT0 ypN0, ypT0/is ypN0, and ypT0/is) for their association with event-free survival (EFS) and OS [6]. Eradicating tumors from both breast and lymph nodes (ypT0 ypN0 or ypT0/is ypN0) had better outcomes associated with improved EFS and OS than did tumor eradication from the breast alone (ypT0/is) in various tumor subtypes, including “luminal A” breast cancer with low histological grade. Therefore, the residual cancer cells in lymph nodes could be important as a prognostic factor.

It is very important to diagnose as clinically lymph node-negative breast cancer before neoadjuvant chemotherapy. There are several established methods of detecting lymph node metastasis, and the choice depends on what clinical practice is preferred or feasible in a given institution. In general, ultrasonography is used as a straightforward diagnostic method. Since ultrasonography providing real-time images can be used in combination with aspiration biopsy cytology, sentinel lymph node biopsy can be omitted by the active use of aspiration biopsy cytology of suspected positive nodes [7]. In daily clinical practice, even in patients diagnosed as having clinically node-negative cancer before NAC, postoperative pathological examination often shows axillary lymph node metastases. The population of ultrasonographically or cytologically node-negative breast cancer may vary in each institute, depending on the aspiration technology used. Therefore, this clinically lymph node-negative population has not been fully studied in terms of predictive or prognostic factors.

In this retrospective study, we investigated the predictive factor and values of ypN+ after NAC for breast cancer in patients with clinically lymph node-negative metastasis.

## Patients and Methods

### Patient selection

**Fig 1:**
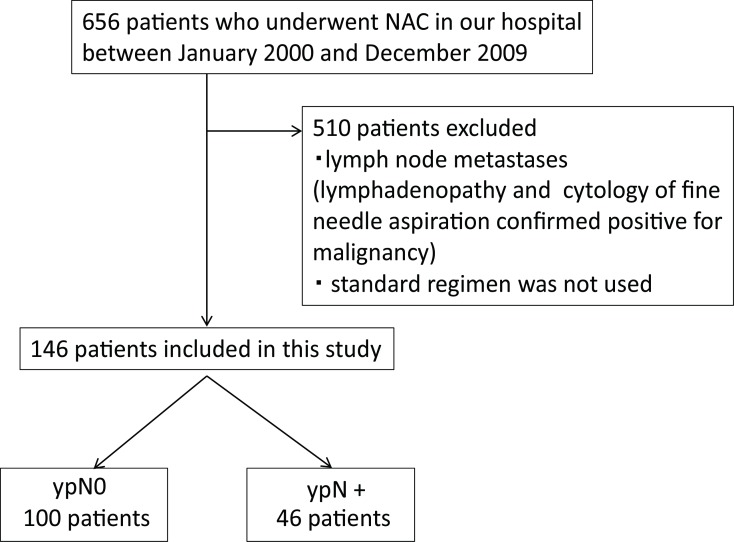
Study profile.

The study profile is shown in Fig 1. The indication criteria for NAC were a tumor diameter of ≥3 cm or axillary lymph node metastasis revealed by preoperative cytodiagnosis. Of 771 patients who underwent NAC in our hospital between January 2000 and May 2009, we included as the subjects 146 patients in whom the standard regimen including anthracycline and taxane was used, and the absence of lymph node metastasis was confirmed before NAC.

Comprehensive consent for the use of specimen materials was obtained preoperatively by written informed consent from all patients participating as subjects in this study. The retrospective study was approved by the Institutional Review Board of the Cancer Institute Hospital of the Japanese Foundation for Cancer Research, and data were collected in compliance with the ethical requirements of our institution.

Biopsy specimens were embedded in paraffin and cut into thin slices with routine histologic sectioning, and immunohistochemical status was evaluated on pre-operation specimens. They were stained with hematoxylin-eosin (HE) and were immunohistochemically examined for estrogen receptor (ER), progesterone receptor (PgR), and HER2. Using HE-stained slices, the histological subtype of cancer and nuclear grade of cancer cells were evaluated according to the general rules for clinical and pathological assessment of breast cancer, as edited by the Japanese Breast Cancer Society [8]. Histological subtype was divided into three groups, i.e., papillotubular carcinoma, solid-tubular carcinoma, and scirrhous carcinoma, based on size and structure of invasive components. These groups were defined follows: papillotubular carcinoma, characterized by large invasive components with papillary structures and/or tubular formations; solid-tubular carcinoma, characterized by large solid invasive components; and scirrhous carcinoma, characterized by small scattered invasive components or trabecular invasive nests with a desmoplastic stroma. Nuclear grade was evaluated using a combination of nuclear atypia and mitotic counts. Nuclear grade was divided into three groups—NG 1, 2, and 3—in order of increasing atypia.

Immunohistochemical assessment of ER and PgR expression was performed using antibodies for ER, clone 1D5 (Dako Japan Inc., Tokyo, Japan) and for PgR, clone PgR636 (Dako Japan Inc.). Positive reactions for ER and PgR were defined as nuclear staining in 10% or more of cancer cells, and negative as staining in less than 10%. Hormone receptor (HR) positivity was defined as showing positivity in ER and/or PgR.

Immunohistochemical detection of HER2 protein was performed using the Hercep Test (Dako Japan Inc.). Expression of HER2 protein was classified into four groups: 0, 1+, 2+, and 3+. In those cases that were 2+, HER2 genetic testing by FISH was performed using a PathVysion HER2-DNA Probe Kit (Abbott Molecular Inc., Des Plaines, IL). Both protein and genetic status were estimated based on the guidelines for HER2 testing in breast cancer, as edited by American Society of Clinical Oncology/College of American Pathologists [9]. HER2 positivity was defined as HER2 protein 3+ or HER2 gene amplification.

Based on the combination of HR and HER2, patients were classified into four subtypes. Subtype definitions were as follows: luminal subtype, HR positive and HER2 negative; luminal HER2 subtype, HR positive and HER2 positive; HER2 enriched subtype, HR negative and HER2 positive; and triple negative subtype, HR negative and HER2 negative.

### Ultrasonography technique and interpretation

In this study, the ultrasound devices used were Toshiba (Japan) Ultrasound Diagnostic System SSA-250A (probe SMA-736SA; center frequency 7.5MHz), SSA-340A(PLF-805ST; 8.0MHz), SSA-350A (PLF-805ST; 8.0MHz), SSA-550A (PLM-805AT; 8.0MHz), SSA-700A (PLT-805AT; 8.0MHz), Hitachi (Japan) Ultrasound Diagnostic System EUB-450 (EIP-L33; 7.5MHz), EUB-2000 (EIP-L53; 7.5MHz), EUB-6000 (EIP-L53; 7.5MHz), EUB-7500 (EIP-L53; 7.5MHz), ALOKA (Japan) Ultrasound Diagnostic System Pro Sound SSD-5500 (UST-5539; 3-11MHz), Pro Soundα10, (UST-5412; 4-13MHz), GE (USA) Ultrasound Diagnostic System LOGIQ 400 PRO (LA39; 8.8MHz), LOGIQ 5 PRO (LA39; 8.8MHz). All axillary US were interpreted by two breast radiologists with 15 years’ experience (over 1000 axillary US breasts reviews per year).

### Definition of preoperatively clinical lymph node-negative metastasis

We have made the definition of clinically lymph node-negative (N0) as follows: first, the ultrasonography before NAC did not show any lymphadenopathy. Second, a cytological procedure confirmed negative study for each patient when ultrasonography suggested lymphadenopathy. Lymphadenopathy was defined as round, hypoechoic nodes, with or without cortical thickening and obliteration of hilum, or > 5 mm.

### SLNB and lymph node pathology

Sentinel lymph nodes were identified using the combined RI-dye method. Sentinel lymph node metastases were diagnosed using intraoperative rapid diagnosis of sections taken at 2-mm intervals for whole nodes. The ypN+ was defined as all single residual cancer cells in lymph nodes.

### Definition of pathological complete response (pCR)

Histological therapeutic effects were determined according to the criteria established by the Committee for Production of Histopathological Criteria of the Japanese Breast Cancer Society [10] and divided into ypT0/is ypN0, ypTinv ypN0, and ypTis/inv ypN+. In our study, ypT0/is ypN0 (No invasive residual in breast or nodes; noninvasive breast residuals allowed), which was used by the MD Anderson Cancer Center trial [11], was defined as pCR. The ypTinv ypN0 (invasive residual in breast and no invasive or noninvasive residual in breast or nodes) and ypTis/inv ypN+ (invasive or noninvasive residuals in the breast, infiltrated lymph nodes) were also used.

### Treatment

Standard chemotherapy with drugs including anthracycline and taxane was administered in this study. The regimens of anthracycline-containing chemotherapy used were FAC (fluorouracil 500mg/m^2^, doxorubicin 50mg/m^2^, cyclophosphamide 500mg/m^2^ every three weeks) and FEC (fluorouracil 500mg/m^2^, epirubicin 100 mg/m^2^, cyclophosphamide 500mg/m^2^ every three weeks). The taxane regimens employed were a weekly paclitaxel (wPAC) regimen at a dose of 80 mg/m^2^, triweekly paclitaxel (3wPAC) regimen at a dose of 175mg/m^2^, and a triweekly docetaxel (3wDOC) regimen at a dose of 75 mg/m^2^. Trastuzumab, which was approved for use as an adjuvant in February 2008 in Japan, was administered in the patients with HER2 positive breast cancer at a loading dose of 8 mg/kg on day 1 of cycle 1 and 6 mg/kg thereafter.

### Statistical analysis

The SPSS ver. 17.0 (IBM Corporation, Armonk, NY, USA) was used for statistical analysis. The DFS and OS rates were analyzed using the Kaplan-Meier method with the log-rank test. Fisher’s exact test was used to evaluate the association between ypN+ and clinical factors. P of <0.05 was considered statistically significant in all instances.

## Results

### Patient characteristics

Patent characteristics of all 146 cases are listed in Table 1. The mean age of the patients was 51, and the tumor diameter before NAC was T1 in 4 patients (2.7%), T2 in 113 (77.4%), T3 in 15 (10.3%), and T4 in 14 (9.6%); T2 or T3 was observed in 128 patients (87.7%). The histological type as established by needle biopsy was invasive ductal carcinoma in 128 (87.7%) and invasive lobular carcinoma in 7 (4.8%). NG1 was observed in 43 (29.5%), NG2 in 38 (26.0%), and NG3 in 40 (27.4%); 81 patients (55.5%) showed NG1 or NG2. In 25 (17.1%) patients, evaluation was impossible due to the special type (14 patients), tissue crush and very small quantity of cancer cells (11 patients). As for the histological therapeutic effects of NAC in breast, 9 patients (6.2%) showed T0/is (pCR) while 137 patients (93.8%) showed Tinv (non-pCR). The subtype was the luminal type in 97 patients (66.4%), the triple-negative type in 30 (20.5%), HER2 type in 13 patients (8.9%), and luminal-HER2 type in 6 patients (4.1%). Ultrasonography revealed axillary lymphadenopathy in 42 (28.8%) of the 146 patients, but fine-needle aspiration (FNA) confirmed no metastasis in these patients. Of these 42 patients, four underwent FNA twice. The pathological examination at the time of surgery showed lymph node metastasis (ypN+) in 46 patients (31.5%). Of the 42 patients in whom ultrasonography revealed axillary lymphadenopathy with no metastasis by FNA, pathological examination at the time of surgery showed lymph node metastases in 14 patients (33.3%).

**Table 1:** Patient characteristics.

	n = 146	
	No.	%
Age		
median	51	
range	19–75	
49>	67	45.9
50≦	79	54.1
Tumor size		
T1	4	2.7
T2	113	77.4
T3	15	10.3
T4	14	9.6
Subtype		
Luminal	97	66.4
Triple negative	30	20.5
HER2	13	8.9
Luminal-HER2	6	4.1
Pathological type		
Invasive ductal carcinoma	128	87.7
Invasive lobular carcinoma	7	4.8
Others	11	7.5
Nuclear Grade		
1	43	29.5
2	38	26.0
3	40	27.4
Special type	14	9.6
Undetermined	11	7.5
Lymphadenopathy before NAC		
yes	42	28.8
no	104	71.2
No. of metastatic lymph node (at the time of surgery)		
0	100	68.5
1–3	36	24.7
4–9	7	4.8
10<	3	2.1
Neoadjuvant chemotherapy		
anthracycline	70	47.9
anthracycline followed by taxane	59	40.4
taxane	17	11.6
Postoperative chemotherapy		
anthracycline	10	6.8
taxane	14	9.6
Histological therapeutic effect		
ypT0/is ypN0	9	6.2
ypTinv ypN0	91	62.3
ypTis/inv ypN+	46	31.5

One hundred thirty-nine patients (95.2%) received anthracycline-containing chemotherapy. and 90 (61.6%) received taxane. FAC and FEC were used preoperatively in 73 (50.0%) and 56 (38.4%) respectively, and postoperatively in 2 (1.4%) and 8 (5.5%) respectively. Paclitaxel and docetaxel were used preoperatively in 66 (45.2%) and 10 (6.8%) respectively, and postoperatively in 5 (3.4%) and 9 (6.2%) respectively. Trastuzumab was used in one (16.7%) of luminal-HER2 type and six (46.2%) of HER2 type.

The mean observation period was 79.7 months, and recurrence was observed in 21 patients (14.4%). There were 12 deaths (8.2%) related to breast cancer.

### Correlation between residual disease and outcome (DFS and OS)

**Fig 2:**
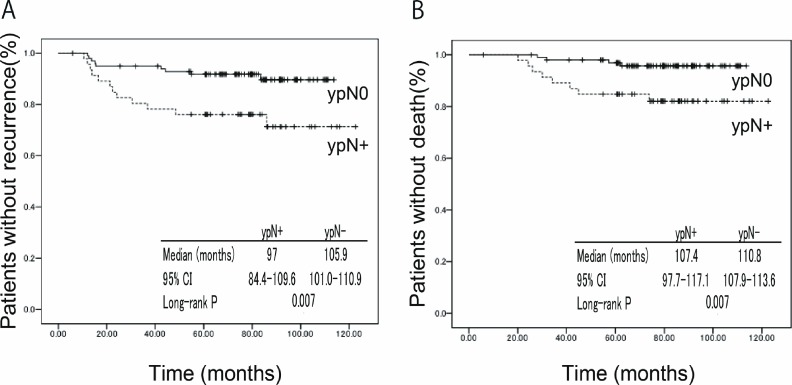
The Kaplan-Meier plots of DFS and OS. (A) The median DFS was significantly longer in patients with ypN0 than ypN+ (105.9 vs 97.0 months, p = 0.007). (B) There was also significant difference in the median OS (110.8 vs 107.4 months, p = 0.007). Abbreviations: ypN0: No invasive residual in nodes, ypN+: invasive or noninvasive residuals in the breast, infiltrated lymph nodes.

The Kaplan-Meier curves of DFS and OS are shown in Fig 2A and 2B. The median DFS was significantly longer in patients with ypN0 (105.9 months; 95% CI 101.0–110.9) than ypN+ (97.0 months; 95% CI 84.4–109.6) (p = 0.007). There was also significant difference in the median OS (110.8 months; 95% CI 107.9–113.6 vs 107.4 months; 95% CI 97.7–117.1, p = 0.007).

### Predictive factors for ypN+ in clinically lymph node-negative breast cancer

Patient characteristics of pathological n+ (ypN+) and n-(ypN0) are listed in Table 2. While there was no significant difference in age, tumor size, pathological type or nuclear grade among the ypN+ and ypN0 groups, significant differences in the histological therapeutic effects (p = 0.039) and subtype (p = 0.029) were observed among the ypN+ and ypN0 groups. As the number of patients with HER2 type and Luminal-HER2 type involved in our study was very small, we analyzed the association between the luminal type and non-luminal type by Fisher’s exact test.

**Table 2:** The predictive factors of ypN+ in N0 breast cancer.

	ypN0 (n = 100)	ypN+(n = 46)	p value
Age			
49>	44 (30.1%)	23 (15.8%)	0.499
50≦	56 (38.4%)	23 (15.8%)	
Tumor size			
T1	3 (2.1%)	1 (0.7%)	0.791
T2	79 (54.1%)	34 (23.3%)	
T3	10 (6.8%)	5 (3.4%)	
T4	8 (5.5%)	6 (4.1%)	
Subtype			
Luminal	61 (41.8%)	36 (24.7%)	0.029
Non-luminal	39 (26.7%)	10 (6.8%)	
Pathological type			
Invasive ductal carcinoma	86 (58.9%)	42 (28.8%)	0.596
Invasive lobular carcinoma	5 (3.4%)	2 (1.4%)	
Others	9 (6.2%)	2 (1.4%)	
Nuclear Grade			
1	27 (18.5%)	16 (11.0%)	0.863
2	28 (19.2%)	10 (6.8%)	
3	27 (18.5%)	13 (8.9%)	
Special type	10 (6.8%)	4 (2.7%)	
Undetermined	8 (5.5%)	3 (2.1%)	
Histological therapeutic effects			
ypT0/is	9 (6.2%)	0 (0%)	0.036
ypTinv	91 (62.3%)	46 (31.5%)	

### Univariate and multivariate analyses of prognostic factors

Univariate and multivariate analyses of prognostic factors are shown in Table 3. The poor prognostic factors for DFS and OS were ypN+ (p = 0.007 and p = 0.007) significantly. And large tumor size before NAC was significantly poor prognostic factor for DFS (p = 0.028), but was not for OS (p = 0.203). Multivariate analysis demonstrated that younger age (49>), large tumor size, NG3 and ypN+ were significant poor prognostic factors (p = 0.020, p = 0.008, P = 0.022 and p = 0.010, respectively). Moreover, ypN+ was the only significant poor prognostic factor for OS (p = 0.022).

**Table 3:** Univariate and multivariate analysis of prognostic factors.

	Univariate analysis		Multivariate analysis			
	P value		DFS		OS	
Factors	DFS	OS	Relative Risk	P value	Relative Risk	P value
Age						
50≦, 50>	0.096	0.143	0.323	0.020	0.356	0.106
Tumor size (pre NAC)						
T1+T2, T3+T4	0.028	0.203	3.588	0.008	2.713	0.119
Subtype						
Luminal, non-luminal	0.641	0.480				
Pathological type						
IDC, ILC, other	0.570	0.398				
Nuclear Grade						
NG1,NG2,NG3,other,UD	0.192	0.577	1.496	0.022	0.971	0.904
Pathological lymph node status						
ypN0, ypN+	0.007	0.007	3.162	0.010	4.086	0.022

## Discussion

Although the challenges of working up a post-NAC breast excision or mastectomy are well documented in the literature, NAC for patients preoperatively diagnosed as having node-negative breast cancer presents several problems. There are several definitions of pCR according to a variety of classifications. Eradication of tumor from both breast and lymph nodes (ypT0 ypN0 or ypT0/is ypN0) has been associated with improved prognosis, and the presence of residual cancer cells in lymph node has been taken to be important as a prognostic factor. We evaluated the predictive factors and value of ypN+ in clinically lymph node-negative breast cancer and showed the median DFS and OS were significantly longer in patients with ypN0 than with ypN+.

Our study revealed two important results. First, one of the predictive factors of ypN+ in clinically lymph node–negative breast cancer was the luminal type. Our study also suggests that tumor diameter before NAC could be a poor prognostic factor for DFS in axillary node-negative breast cancer. The majority of ER-positive HER2-negative luminal breast cancer grows slowly, with a low apoptosis rate and low genetic instability [12]. Foulkes et al. proposed that slow-growing tumors can cause heterogeneity within the tumor, and many tumor cells acquire the ability for distant metastasis. Loo et al. reported changes in tumor diameter in MRI during NAC [13]. In their study, massive tumor regression after chemotherapy were observed only in the triple-negative and HER2-positive tumors, and the presence of residual tumors in surgical specimens after NAC in ER-positive/HER2-negative tumors was not correlated with the change in largest diameter of late enhancement during NAC. They commented that this striking observation might be explained by the heterogeneous appearance in the ER-positive/HER2-negative group. Therefore, heterogeneity in the luminal type of breast cancer might be involved in residual cancer cells in breast and lymph nodes.

The second is the false-negative rate of ultrasonography before NAC for regional lymph node metastasis in clinically lymph node-negative breast cancer. We evaluated regional lymph nodes for size and morphology using sonographic criteria or sonographically guided fine-needle biopsy. In 46 (31.5%) of 146 patients diagnosed with node-negative breast cancer before NAC, pathological examination at the time of surgery showed lymph node metastases.

Multiple methods can be used to establish the positivity of regional lymph nodes. To diagnose regional lymph node metastasis, ultrasonography is most widely used in clinical practice. The diagnostic criteria of axillary lymph node metastasis using ultrasonography mostly include a short axis length ≥ 5 mm. Alzarez et al. reviewed 16 studies, noting a sensitivity and specificity of diagnosis according to the node size of 48.4–87.1 and 55.6–97.3%, respectively, and a sensitivity and specificity of diagnosis according to morphological criteria of 26.4–75.9 and 88.4–98.1%, respectively, in patients with non-palpable axillary lymph nodes [14]. Using sonographic criteria or sonographically guided fine-needle biopsy, our study showed the false negative rate of ultrasonography was 31.5%, which was comparable to the 37.1% that reported by Alzarez et al.

Possible causes of the high false-negative rates, both in this study and in the review, include the following. The first is a characteristic of subjects enrolled in this study. All patients with clinically node negative breast cancer in our study were at high risk and needed neoadjuvant chemotherapy. Therefore, the pathological examination at the time of surgery might be expected to show a higher positive rate for lymph node metastasis. The second is quality of the ultrasonograph. During this study, the ultrasonography that we used clinically did not have high enough resolution. Recently, we have been using second-look targeted ultrasound (2^nd^ Look US), and the resolution of ultrasonography in our hospital has improved greatly.

In our study, the definition of pCR was determined according to the criterion established by M.D. Anderson trial: no invasive residual in breast or nodes; noninvasive breast residuals were allowed (ypT0/is ypN0). According to this criterion, the pCR rate was 6.2% in all patients in our study, which is lower than reported in the literature. Recently it was reported that the prognostic impact of pCR varied among tumor subtypes classified by the status of HR and HER2. In our study, the pCR rate according to subtypes were 2.1% (2/97) in the luminal type, 16.6% (5/30) in the triple negative type, 15.4% (2/13) in the HER2 type, and 0% (0/6) in the luminal-HER2 type. From our institute, Kobayashi et al. reported that the pCR rate defined as ypT0/is ypN0 was 8.5%, which was almost equal to the rate in our report [15]. The discrepancies among institutions and pathologists might be caused from the differences in examination methods of surgical specimens or the ability to determine pathological diagnoses. In pathological diagnosis, a high level of skill is required to recognize tiny amounts of cancer in a surgical specimen after treatment. In our institute, we examined surgical specimens extensively and thoroughly. All slices were reviewed by the same expert pathologist, who is specialized in breast cancer pathology with over 20 years’ experience (over 1000 pathological reviews per year). Another reason for the difference might be that trastuzumab therapy was not used in the majority of HER2+ patients because it was not approved as preoperative chemotherapy during this study period in Japan.

The limitation of the present study is that there was no information about proliferative marker indices such as Ki-67. Luminal A breast cancer was defined as positive for ER and negative for HER2 in the CALGGB9344 trial [16], positive for ER and/or PgR with a low Ki67 labeling index (cut-off of 13%) in the BCRG01 trial [17], and positive for ER with a low Ki67 labeling index (cut-off of 20%) in the PACS01 trial [18]. In addition, the St. Gallen International Conference 2011 proposed that breast cancer positive for ER and/or PgR, negative for HER2 with a Ki67 labeling index of less than 14%, should be defined as luminal A breast cancer [19]. In the St. Gallen International Conference 2013, the definition of luminal A breast cancer was proposed as ER and PgR positive, HER2 negative and Ki-67 “low.” No absolute values of a cut-off between “high” and “low” were suggested during this conference; locally useful cut-off points are adopted in daily practice to distinguish between luminal A and B [20]. Because the definition of luminal A breast cancer has not been established, and the methodology of Ki67 has not been standardized [21], we used nuclear grade as a proliferative marker index in this analysis.

Other limitations of the present study are the following. It is a retrospective study from a single institute. We can now use newer treatment regimens. Although we use both weekly paclitaxel regimens at a dose of 80 mg/m^2^ and triweekly paclitaxel regimens at a dose of 175 mg/m^2^, we typically use weekly paclitaxel regimens based on the ECOG1199 trial [22]. Trastuzumab therapy was not included in the patients of this study because it was not a standard preoperative treatment during this period in Japan.

In many recent studies, pCR rates associated with NAC have been regarded as a surrogate endpoint for DFS and OS. As noted earlier, the US Food and Drug Administration has recently attached great importance to the pCR rate in deciding whether to approve new anti-cancer agents. We reconfirmed ypN+ was the significant poor prognostic factor for DFS and OS. Moreover, we revealed that the luminal type could be predictive of pathological lymph node metastases, suggesting high heterogeneity inside the tumors and involvement of scattered remaining cells in the resistance to therapy. This finding is very informative because the effects of the current standard therapy, i.e., sequential therapy with anticancer agents, anthracycline and taxane, might be limited in pursuing higher pCR rates in highly heterogeneous luminal breast cancer. We must construct a strategy that leads to eradication of tumors from breast tissue as well as from the lymph nodes. Therefore, it is crucial to identify the clinicopathological factors that may influence metastases to the lymph nodes in clinically lymph node-negative breast cancer before NAC.

In conclusion, 31.5% of patients preoperatively diagnosed with lymph node-negative breast cancer before NAC showed lymph node metastasis at the time of surgery. The prognosis of clinically lymph node-negative breast cancer depended on ypN+, which was associated with ypTinv and luminal subtype.

## Supporting Information

S1 Table Clinical information for neoadjuvant chemotherapy dataset. (XLSX)
